# “*Pisando Fuerte*”: an evidence-based falls prevention program for Hispanic/Latinos older adults: results of an implementation trial

**DOI:** 10.1186/s12877-019-1273-1

**Published:** 2019-09-18

**Authors:** Maria Mora Pinzon, Shannon Myers, Elizabeth A. Jacobs, Sherri Ohly, Militza Bonet-Vázquez, Marcia Villa, Al Castro, Jane Mahoney

**Affiliations:** 1 Department of Medicine, Division of Geriatrics and Gerontology, School of Medicine and Public Health, University of Wisconsin – Madison, Madison, WI USA; 2 Community Academic Aging Research Network (CAARN), University of Wisconsin – Madison, 610 Walnut St. Office 330E, Madison, WI 53704 USA; 3 Wisconsin Institute for Healthy Aging, Madison, WI USA; 4 Division of Primary Care and Value Based Health, Departments of Internal Medicine and Population Health, University of Texas at Austin Dell Medical School, Austin, TX USA; 5 JCS Consulting Solutions, Milwaukee, WI USA; 6 Medical College of Wisconsin, Milwaukee, WI USA; 7 United Community Center, Milwaukee, WI USA

**Keywords:** Implementation, Fall prevention, Hispanic/Latinos, Hispanic, Community-dwelling, Older adults

## Abstract

**Background:**

We previously developed *Pisando Fuerte (PF)*, a linguistically and culturally appropriate version of “Stepping On”, an evidence-based fall prevention program building on self-efficacy and adult learning principles. The purpose of this study is to describe the implementation of *PF* at two community organizations in Wisconsin.

**Methods:**

*PF* consisted of 2 h sessions delivered in Spanish over the course of 8 weeks by two trained leaders, at two community sites in Wisconsin. Participants identified strategies for falls prevention and practiced progressive balance and strength exercises. The RE-AIM framework guided the mixed-methods evaluation. Falls Behavioral Risk Scale (FaB) (*Outcomes*), and uptake of protective behaviors (*Individual Maintenance*) were evaluated 6 months after completion. Fidelity of delivery (*Implementation*) was evaluated by an independent assessor for three sessions at each site using a-priori criteria based on key elements of Stepping On.

**Results:**

Twenty-four Hispanic/Latino individuals, whose primary language is Spanish, were enrolled in two workshops. The mean age was 70.5 years; 71% were female, and five reported a fall in the year prior. *Outcomes:* There was a non-statically significant decrease in the number of falls per person [RR: 0.33 (95%CI: 0.096–1.13)] at 6 months. There was a statistically significant improvement of the mean Falls Behavioral Risk Scale (FaB) (baseline = 2.69 vs. 6-months post-intervention = 3.16, *p* < 0.001). *Adoption:* Barriers to adoption included leader training in English, time to identify Spanish-speaking guest experts, and time to prepare for each session. *Implementation:* Satisfactory fidelity of delivery was achieved in 69% of the elements; fidelity lapses were more common in the use of adult learning strategies and programmatic aspects. Eighty eight percent of participants completed the program, and 95% of them adequately demonstrated the exercises. *Maintenance*: At 6 months, 57.9% of participants continued doing exercises, 94% adopted safer walking strategies, and 67% executed at least one home safety recommendation. These results are similar to those seen in the original Stepping On program.

**Conclusions:**

Our study shows good fidelity of delivery with implementation of “*Pisando Fuerte*”. Pre-post data demonstrate a significant reduction in falls behavioral risk among Hispanic/Latino participants, similar to results with “Stepping On”.

**Trial registration:**

ClinicalTrials.gov, NCT03895021. Registered March 29, 2019.

## Introduction

In the United States (US), every hour, three older adults die as a result of a fall, and by 2030 this number is expected to increase to seven older adults [1]. The risk of falling is similar across race and ethnicity [2], and although the age-adjusted death rate due to falls is higher for whites, the age-adjusted death rate for Hispanic/Latinos seniors has been climbing over the last few years [3]. This is concerning because the older adult Hispanic/Latinos population in the US is growing exponentially [4], and there is a dearth of evidence-based fall prevention programs designed for Hispanic/Latino seniors.

Risks factors for falls include certain medications, vision impairment, gait and balance impairments, muscle weakness, inadequate footwear, environmental hazards, and chronic conditions (e.g. arthritis, dementia) [5, 6]. Some evidence-based fall prevention programs that target risk factors for falls or fear of falling are available in Spanish, and they have been shown to reduce fear of falling or risk factors for falls, resulting in improve physical activity, balance, and/or lower extremity strength. However, they have not shown a decrease in falls among Hispanic individuals [7–10].

The process to select one fall prevention initiative to implement usually involves a needs assessment process at the community/organization level, which takes into account the cost of the delivery, preferences of participants, capacity of staff, among others. According to Hanlin et al., seniors in Hispanic communities in Milwaukee (Wisconsin, US) would prefer a multi-faceted program that combines exercise with education in Spanish, and with the participation of a healthcare provider [11]. *Stepping On* is a multifaceted intervention with similar characteristics to those described as desired by Hispanic communities in Wisconsin. It has been documented to decrease the risk of falls among Australian older adults by 31% [12–14], and is available in English in 22 states within the US.

Given the growing population of Hispanic older adults, and the need to provide a culturally and linguistically tailored multifaceted fall prevention program for Hispanic seniors, we adapted *Stepping On* to develop *Pisando Fuerte* for use with Hispanic/Latinos older adults whose primary language is Spanish.

The primary objective of this paper is to describe the implementation of *Pisando Fuerte* at two community organizations in Wisconsin using the RE-AIM framework [15], which includes a characterization of the reach, adoption, pre-post outcomes, implementation fidelity and costs, and maintenance of behavior change by individuals. The secondary objectives include to: 1) identify the barriers and facilitators for adoption and implementation from the perspective of leaders and program coordinators, and 2) describe the effects of the program in terms of participants’ uptake of behaviors that have been shown to be effective in preventing falls: practice of strength and balance exercises, and protective health behaviors (e.g. avoiding hazardous conditions, using adequate footwear).

## Methods

### Study design

A single arm clinical trial was performed. Workshop participants were recruited by leaders and staff from the community organizations that participated in the study. After screening was completed by the leader, those that complied with the following characteristics were invited to participate in the program: Self-Identification as Hispanic or Latino, 65 years old or more, living independently, had fallen in the past year or reported a fear of falling, without history of dementia. Individuals that needed help of another person to ambulate or used a standard walker to ambulate indoors were excluded. Each participant completed a written informed consent to participate in the research study, according to the regulations established by the University of Wisconsin – Health Sciences Institutional Review Board, which approved this study.

### Settings

Two community organizations that serve Hispanic/Latino communities in Wisconsin were invited to participate in 2014–2015. Site 1 was a senior center with experience providing fitness programs and health promotion programs for English and Spanish-speaking older adults. The workshop was held at a multi-purpose facility that hosts community activities year round. The leaders for site 1 were identified by the organization and served as co-leaders: a bilingual physical therapist (non-native Spanish-speaker) with experience facilitating health promotion programs; and the outreach and program planner for the Latino Senior Program, who was a native Spanish-speaker with experience working with older adults, and previous experience delivering group-based health education classes to seniors.

Site 2 was a community organization that delivers health education programs and other social services at senior housing facilities. The workshop was held within an apartment complex that has primarily Hispanic senior residents. The lead leader was a bilingual coordinator for evidence-base programs at the Wisconsin Institute for Healthy Aging (non-native Spanish-speaker), who had several years of experience delivering self-management programs and facilitating group interventions. The peer-leader was the senior housing coordinator, who was a native Spanish-speaker, without experience facilitating groups.

### Adaptation of *Stepping On* to *Pisando Fuerte*

The process to develop *Pisando Fuerte* and its pilot study were performed in 2011–2012 [16]. A community-academic advisory board was convened to recommend initial modifications to the *Stepping On* curriculum. The Advisory board was formed by providers of community-based programs for Hispanic/Latinos older adults, as well as experts in fall prevention, health promotion in Hispanic/Latinos populations, language translation, and kinesiology. The Advisory Board also included Hispanic older adults whose primary language was Spanish.

The adaptation was done using a systematic, rigorous process, following best practices in cultural adaptation [17–19]: the inclusion of a variety of stakeholders in the process; using focus groups and interviews to assess population needs and important contextual factors; modifications to the program were performed to increase acceptability, engagement and adherence to the program; several iterations of the program were performed to allow for refinement of adaptations without compromising effectiveness; and comprehensive evaluation of the program at each iteration to assess the efficacy of the modification in improving acceptability, engagement and adherence.

For the pilot study, only the handouts for participants were translated to Spanish, leaders had to translate information and examples as they delivered the program [16].

Based on results of the pilot, the following changes were implemented to facilitate adoption and implementation by community organizations: 1) Information about the content of the sessions in the leader manual was translated to Spanish, 2) an extra session was added to provide more time for interaction, and 3) pre-qualifications of leaders were expanded to allow health educators and community health workers to train as leaders.

The resulting program (*Pisando Fuerte*) maintains all but two of the key elements of *Stepping On* [20]: 1) Use of weights for leg exercises was changed to optional rather than required, in response to the community advisors’ consensus that most of the older Hispanic/Latinos participants would not comply with using weights, due to monetary and cultural barriers. **2)** Background of the leaders. For *Stepping On*, potential leader candidates must comply with three characteristics that have been previously identified as key to success [21]; however, for *Pisando Fuerte*, given the dearth of Spanish-speaking health professionals, to increase adoption we relaxed the requirement that the program leader be an allied health professional or fitness expert, so that the potential candidate could also be a community health worker or other professional working with Hispanic/Latino older adults. Table 1 summarizes the main differences between *Stepping On* and *Pisando Fuerte*.

**Table 1:** Comparison between Pisando Fuerte and Stepping On

	Stepping On	Pisando Fuerte	Rationale for adaptation
Language	English	Spanish	Hispanic/Latino older adults preferred Spanish.
Reading level of program	8th Grade	Leader’s manual: 8th Grade Handouts: 3rd Grade	Hispanic/Latino older adults in the study had low educational attainment.
Requirements to be a leader	(1) Background as an allied health professional (RN, PT, PTA, OT, social worker) or fitness expert; (2) Experience facilitating an adult self-management program; (3) Professional experience working with older adults [21].	(1) Professional experience working with Hispanic/Latino older adults (2) Background as allied health professional, fitness expert, community health worker, health educator, promotor(a), or equivalent experience.	Recruiting for leaders was difficult, few active health professionals are from a Hispanic/Latino background AND fluent in Spanish. Community organizations did not have staff with the required characteristics, and they could not assure sustainability of the program without the changes.
Number of sessions	7 session (2 h each)	8 sessions (2.5 h each)	During pilot, sessions lasted longer than expected.
Sessions^a^	- Strength and balance exercises - Use of assistive devices - Preventing falls indoor/outdoor - Vision & footwear - Medications & sleep habits - Bone Health	- Strength and balance exercises - Use of assisted devices - Preventing falls indoor/outdoor - Vision & footwear - Medications & sleep habits - Bone Health - *How to talk to your doctor? – Modified*	The session about “how to talk to your doctor” was modified per stakeholder input to add more emphasis on the importance to voice concerns to health professionals; and how to address barriers.
Use of weights for exercise	Mandatory	Optional	Cost of the weights is prohibitive to the organizations and participants, and cultural barriers would reduce use.
Guest Experts	In person visits from guest experts.	If guest experts are not fluent in Spanish, leaders could use any of the following: - Invite an English speaker guest expert and use an interpreter to deliver the information - Use pre-recorded videos with Spanish-speaking guest experts, and bring an English speaker for questions & answers, with an interpreter to facilitate discussion.	It was a challenge to identify and bring bilingual guest experts to participate in the sessions. These modifications assure that the key elements of the program are maintained.
Other		- Inclusion of Hispanic/Latinos cultural values - Created a resource book for leaders on how to find resources in your community. - Supporting materials (e.g. Presentations, Handouts) included more pictures, and stories were adapted to be culturally appropriate - Participants were asked to share information with family members and family members were invited to the last session	These changes assured acceptability of the program by increasing fun, build trust in leaders, increase involvement of family members, and facilitated participation during sessions.

^a^The order of the sessions is not described in this table. For *Pisando Fuerte* the order was modified to fit the additional session and extra time

### The intervention

*Pisando Fuerte* is a multifaceted community-based program that is provided in a small-group learning environment to improve falls prevention self-efficacy, encourage adoption of preventive behaviors, and provide progressive strength and balance exercises for older adults whose primary language is Spanish. *Pisando Fuerte* consists of 8 sessions, each one of 2.5 h, delivered on a weekly basis, by one trained leader and one peer-leader, followed by a booster session at 3 months. Several of the sessions involve the use of guest speakers, who provide a short lecture to participants, followed by time for questions. The guest experts are not compensated for their time, and they represent the following specialties: physical therapists, pharmacist, ophthalmologist or optometrist, community safety expert or law enforcement.

*Pisando Fuerte* leaders took the 3 day English language Stepping On training, offered by the Wisconsin Institute of Healthy Aging [22], followed 1 month later by a 1-day cross training in English and Spanish on *Pisando Fuerte,* which covered the differences between the programs, important considerations in Hispanic/Latinos culture, and how these may affect implementation of the workshop and participant uptake of health behaviors.

### Evaluation framework

The RE-AIM framework was used to evaluate the implementation of the program [15]. This framework evaluates five domains (Reach, Effectiveness, Adoption, Implementation, Maintenance) that are important in the translation of health behavior interventions to practice. Because our study was not powered to assess the effectiveness of the adaption, we used pre-post health outcomes as a surrogate of this domain. Health outcomes were assessed 6 months after the intervention, and compared with baseline assessments. Table 2 defines the domains and associated metrics as applied to this study.

**Table 2:** Re-Aim domains and definitions

Domain	Definition for Pisando Fuerte	Measurement
Reach	Number and characteristics of individuals willing to participate in a program.	Number of participants in the program compared to the number of individuals invited to participate. Overall sociodemographic characteristics of participants.
Outcomes	The health outcomes associated with the intervention	Number of falls per person during 6 months post intervention compared to 6 months pre intervention, collected during interviews, and corroborated with the use of tracking calendars that were submitted during the study period. The Timed Up and Go (TUG) [23] and Shortened Fall Behavioral Scale (FaB) scale [24] were used as intermediary outcomes; these were measured at baseline and 6 months post intervention.
Adoption	Characteristics of adopting organizations, and barriers and facilitators to adoption by the organizations.	Content analysis of semi-structured interviews of leaders, coordinators and administrators after the workshops were completed.
Implementation	At the organization level, it refers to the fidelity of delivery of the intervention, time/cost to implement the program.	Fidelity of delivery – Evaluation was performed at sessions 1, 3, and 7, by a trained expert, who used a pre-defined checklist created using the key elements of Stepping On. Time/cost to implement the program according to budgets and hourly invoices submitted by adopting organizations.
	At the individual level, it refers to the use of the intervention by participants	Fidelity of enactment – the degree to which the participants apply the skills learned in their daily life [25]. It was assessed at completion of the program and 6 months post intervention using content analysis of semi-structured interviews of participants and demonstration of exercises. Workshop completion was defined as attending 6 out of 8 sessions.
Maintenance	At the organization level refers to the capacity to keep implementing the program	N/A – Because *Pisando Fuerte* was only available for research purposes, we could not assess the maintenance of the program.
	At the individual level, refers to the long-term fidelity of enactment of the health behavior of interest.	Maintenance is the degree to which the participants apply the learned skills long-term in their daily life [25]. Measured using content analysis of semi-structured interviews of participants 6 months post completion.

### Data collection

Participants completed a survey at baseline that included demographics, history of falls in the past 6 months, and the Shortened Fall Behavioral Scale (FaB) scale, a 24-item validated tool that assesses the presence of protective behaviors for falls in older adults using a scale between 0 (not at all) and 4 (very much) [24, 26]. Functional mobility was assessed by trained evaluators using the Timed Up and Go (TUG) [23, 27], using the following instruction: *“Levántese de la silla, camine 10 pies a un paso cómodo, gire, y regrese a la silla a sentarse otra vez”,* which translates to: “Stand up from the chair, walk 10 feet using a comfortable pace, turn around, and walk back and sit down.” The measure was performed twice at each assessment, but only the second measure was used for analysis.

Fidelity of delivery was assessed at sessions 1, 3 and 7, using pre-established checklists that were created based on the key elements of Stepping On [20]. Additional items incorporating cultural values were developed based on previously published literature [28–30], and reported under the category: cultural adaptation elements (Table 3). To complete these fidelity forms, trained observers marked “Satisfactory”, “Not Satisfactory”, or “Not Done” for each element listed, and commented on all items that were not satisfactory. Leaders were coached after fidelity checks about deficiencies observed during evaluation.

**Table 3:** Elements of cultural adaptation evaluated in fidelity checks

Session 1	
• Leader acknowledged, appreciated use of, and/or incorporated spiritualism, *machismo*, *marianismo*, and *familismo* to enhance rapport.	
• Leader attempted to ensure that cultural values (spiritualism, *machismo*, *marianismo*, *familismo*) did not get in the way of self-management.	
• Leader built on culture and cultural values to help motivate participants.	
• Family was integrated into the session (e.g. in discussion, stories, etc.)	
Session 3	
• Content appeared to be at a level that was right for participants.	
• Leader builds on high value of relationships.	
• Leader incorporated spirituality appropriately.	
• Leader used opportunities related to machismo and marianismo to promote the *Pisando Fuerte* curriculum.	
• Leader incorporated fatalismo to promote self-management.	
• Leader builds on high value placed on *familismo*.	
• Leader built on culture and values to help motivate participants.	
Session 7	
• Leader acknowledged, appreciated use of, and/or incorporated spiritualism, machismo, *marianismo*, and *familismo* to enhance rapport.	
• Leader attempted to ensure that cultural values (spiritualism, machismo, *marianismo*, *familismo*) did not get in the way of self-management.	
• Leader built on culture and cultural values to help motivate participants.	
• Family was integrated into the session (e.g. in discussion, stories, etc.)	

In the first week after participants completed the program, they were asked to demonstrate the exercises learned during the program, and they were evaluated on technique and knowledge of the exercise. Semi-structured interviews were performed to gather their impressions of the program, uptake of exercise and protective behaviors, and any consequences of participation (Measures of Implementation and Maintenance – Table 2).

Participants received monthly calendars to track falls during the program and the 6 months after completion of the program. Participants returned the calendars to the study office in postage paid, pre-addressed envelopes. Participants who did not return calendars were contacted by phone to obtain falls data.

At 6-months, participants retook the FaB scale and TUG test (Measure of Outcomes – Table 2) and participated in a semi-structured interview either in-person at the community organization or over the phone. The purpose of the interview was to explore the participant’s satisfaction with the program, the changes that they had noted since the completion of the program, their current habits related to uptake of the exercises and fall protective behaviors (maintenance of the program on the individual level), and any unintended consequences because of their participation. Bilingual research staff performed the interviews in Spanish, and then translated them to English for analysis. Semi-structured interviews of leaders were performed after the program was completed with the goal of capturing their experiences, their perceived barriers and facilitators for adoption, and suggestions for future revisions of the program. All interviews were transcribed.

Leaders and administrators tracked number of hours used in the preparation and delivery of the program, and the costs of snacks and other materials.

Study data were collected and managed using REDCap electronic data capture tools hosted at the University of Wisconsin-Madison, School of Medicine and Public Health [31]. REDCap (Research Electronic Data Capture) is a secure, web-based application designed to support data capture for research studies, providing: 1) an intuitive interface for validated data entry; 2) audit trails for tracking data manipulation and export procedures; 3) automated export procedures for seamless data downloads to common statistical packages; and 4) procedures for importing data from external sources.

### Data analysis

Descriptive statistics are reported for participant demographics, FaB, TUG, fidelity measures (delivery, enactment) and maintenance. Wilcoxon Rank Tests (Non parametric) were performed as appropriate to compare continuous variables in univariate analyses (TUG, FaB). A logistic regression with binomial distribution accounting for within-subject correlations assessed intervention effect on falls. All *p* values were 2-tailed, and *p* < 0.05 was the criterion for statistical significance.

Content analysis was used to analyze the transcripts of the interviews [32, 33]; the structure for the categories was identified initially by domains included in the RE-AIM framework [15]. Secondly, we used inductive content analysis, to identify emerging codes and themes, and group them into three large categories: leaders, barriers, other concepts. Additionally, to evaluate fidelity of enactment and maintenance of exercises and fall protective behaviors, responses to these questions were also coded as follows: Yes = if participant reported taking up the desired behavior; No = if participant did not take up the desired behavior. One of the authors performed the primary analysis, and reviewed the results with the research team during the analysis phase to ensure rigor.

Fidelity of delivery assessments were analyzed separately for each site to identify implementation differences, and then analyzed together to identify common elements.

Cost per participant was calculated by adding the costs associated with the delivery of the program and dividing by the number of participants that completed the study. The information was collected from invoices submitted by the community organizations during the study. We excluded the costs of the training and the time used for research activities (e.g. Institutional Review Board training, data collection exclusively for research purposes).

## Results

### Reach

Workshop leaders approached 29 individuals at two community organizations, all of whom fitted the inclusion and exclusion criteria. Out of these, 24 (82.8%) agreed to participate, 15 at Site 1 (93.8% of screened individuals at Site 1), and 9 at Site 2 (69% of screened individuals at Site 2). Mean age was 70.5 years; 71% were female, and five (21%) had reported a fall in the year prior to the program. Table 4 shows the demographic characteristics of participants.

**Table 4:** Sociodemographic characteristics of study participants

Characteristics	Site 1 (*N* = 15)	Site 2 (*N* = 9)	Total (*N* = 24)
Age [Median (Range)]	70 (60–77)	65 (61–95)	70.5 (60–95)
Sex			
• Male	13%	56%	29%
• Female	87%	44%	71%
Marital Status			
• Married	33%	44%	38%
• Single	20%	11%	17%
• Divorced	27%	33%	29%
• Widowed	20%	11%	17%
Education			
• No formal education	7%	11%	8%
• Primary	27%	56%	38%
• Middle/High school	40%	33%	38%
• Technical/College	20%	–	13%
• Graduate	7%	–	4%
Birth Place			
• United States	7%	56%	25%
• Other Country	93%	44%	75%
Cultural roots origin			
• Colombia	27%	–	17%
• Mexico	33%	67%	46%
• Puerto Rico	7%	33%	17%
• Other	33%	–	20%
Race			
• White	40%	56%	46%
• Other	47%	44%	46%
• Not Reported	13%	–	8%
Communication abilities			
• Speaks a language other than Spanish	33%	33%	33%
• Difficulty reading Spanish	13%	78%	37%
Household Size			
• 1	13%	56%	29%
• 2	33%	33%	33%
• 3 or more	53%	11%	38%
How frequently do you see a family member?			
• Daily	50%	20%	29%
• Weekly / Bi-weekly	50%	20%	29%
• Monthly	–	40%	29%
• Not applicable	–	20%	14%
Distance from Workshop			
• Nearby/Walking distance	7%	78%	33%
• 5-10 miles	13%	–	8%
• Don’t Know/Not reported	80%	22%	58%
Transportation Mode to workshop			
• Friend/Family drives	7%	–	4%
• Participant drives	13%	11%	13%
• Other	7%	–	4%
• Public transportation	–	11%	4%
• Taxi	73%	–	46%
• Walking	–	78%	29%

### Outcomes at 6 months

Eighteen participants (75%) completed the 6-months evaluation, those that completed the program were more likely to: have at least middle school education, have been born outside of the United States, and have no difficulties reading in Spanish. Using a pre-post design, comparing to the number of falls in the 6 months prior to the program, participants in the study had a non-significant decrease in the number of falls per person, RR: 0.33 (95%CI: 0.096–1.13). Regarding the TUG results, there was a non-significant increase in the length of time required to complete the task (12.5 vs. 13.4 s), mean difference + 1.3 s, *p* = 0.07. The FaB scale improved significantly from an average of 2.69 at baseline (SD: 0.41), to 3.16 (SD: 0.31), *p* < 0.0001 at 6 months post completion, which is consistent with increased uptake and maintenance of fall protective behaviors.

### Adoption

Three themes were identified in the interviews of leaders, coordinators and administrators regarding the barriers for facilitators and organizations to adopt the program: 1) Issues with translation 2) Language of leader training 3) Difficulty in finding Spanish-speaking guest experts.

One of the barriers identified in several of the interviews was *issues with the translation of the leader manual*, which was characterized by use of words and references that were not native to participants (e.g. Chilean Spanish vs. Mexican Spanish), and the mismatch between the language level of the leader manual (8th grade) and the level of spoken Spanish of participants (3rd grade). A consequence of these issues was that leaders required more time than expected to prepare and deliver each workshop.

*“Language in the Pisando Fuerte book was a challenge. Different types of Spanish. The translation wasn’t as conversational, more academic. Leader helped by making the language more appealing to participants.”*

Additionally, the training of leaders and some parts of the leader manual (preparatory materials before starting the workshop) were in English, which made it more difficult for leaders to learn and implement the program.

*“Training was difficult—wanted Spanish-speaking–training, but it was done in English. Big mistake”*

*“Materials were not in Spanish. Infrastructure instructions (setting up class) should be in Spanish.”*

All the coordinators stated that it was challenging to identify guest speakers that were fluent in Spanish, even in a large city like Milwaukee, with a higher density of Hispanic/Latinos professionals.

*“... It was difficult getting guest speakers. Finding Spanish-speaking people with expertise for the specific time offering class is a challenge. They are in high demand because there are so few of them. They had to use an English-speaking pharmacist because no Spanish-speaking were available.”*

Interviews also explored the *facilitators* for adoption. The common theme was that the flexibility of the program was very important, because it provided an opportunity to connect with participants and adjust the program to their needs.

*“It was rigid but there was some flexibility. Seniors were talking during class and learning without an emphasis on the leader getting through every single page.”*

*“Leaders enjoyed having freedom to work with the class as they see fit; not change curriculum.”*

### Implementation

#### Organization level

Excluding the time required for training, the time invested by leaders preparing and delivering the classes ranged from 4.6 h to 18.6 h per week. Calculating the salaries for leaders at $31.15 per hour, the total amount spent in salary support per workshop was $4411 or an average of $2206 per leader. Additional costs of the program include snacks, and copies/flyers for participants; the cost associates with this ranged from $17.60 to $31.25 per class. After including the cost of supplies and salaries, the total cost per participant enrolled in the program was $367.63. Additional costs related to training and research activities (e.g. Institutional Review Board training, data collection, evaluation of results, and feedback on materials) were not included in this study.

Table 5 shows the results of the evaluation of fidelity of delivery. Overall, each element occurred 92% of the time, but when considering only the elements that were delivered in a satisfactory manner, the overall fidelity decreased to 69%, with lower adherence seen in cultural adaptation, program aspects (e.g. processes specific to the delivery of the program: snacks and beverages available at sessions, use of the medication record card, handouts, group size), and adult learning techniques (e.g. elements used to promote engagement and class participation: plain language, invite feedback, use of the prevention framework) [20]. Additionally, at site 2, one of the sessions was cancelled, and the booster session was not performed due to organizational factors.

**Table 5:** Fidelity of delivery according to evaluation categories

Category	Site 1 n (%)	Site 2 n (%)	Overall^a^ n (%)
Adult learning *(33 items)*			
Satisfactory	25 (76%)	20 (61%)	45 (68%)
Not Satisfactory	1 (3%)	6 (18%)	7 (11%)
Not Done	7 (21%)	7 (21%)	14 (21%)
Program aspects *(19 items)*			
Satisfactory	14 (74%)	10 (53%)	24 (63%)
Not Satisfactory	–	5 (26%)	5 (13%)
Not Done	5 (26%)	4 (21%)	9 (24%)
Exercise elements *(11 items)*			
Satisfactory	9 (82%)	10 (91%)	19 (86%)
Not Satisfactory	1 (9%)	–	1 (5%)
Not Done	1 (9%)	1 (9%)	2 (9%)
Upgrading Exercise *(7 items)*			
Satisfactory	5 (71%)	6 (86%)	11 (79%)
Not Satisfactory	–	–	–
Not Done	2 (29%)	1 (14%)	3 (21%)
Knowledge of the leader regarding falls *(2 items)*			
Satisfactory	2 (100%)	1 (50%)	3 (75%)
Not Satisfactory	–	1 (50%)	1 (25%)
Not Done	–	–	–
Group Leader’s role *(17 items)*			
Satisfactory	16 (94%)	10 (59%)	26 (76%)
Not Satisfactory	1 (6%)	5 (29%)	6 (18%)
Not Done	–	2 (12%)	2 (6%)
Elements of peer co-leader role *(2 items)*			
Satisfactory	2 (100%)	1 (50%)	3 (75%)
Not Satisfactory	–	1 (50%)	1 (25%)
Not Done	–	–	–
Cultural Adaptation *(17 items)*			
Satisfactory	12 (71%)	7 (41%)	19 (56%)
Not Satisfactory	–	–	–
Not Done	5 (29%)	10 (59%)	15 (44%)
Overall *(108 items)*			
Satisfactory	85 (79%)	65 (60%)	150 (69%)
Not Satisfactory	3 (3%)	17 (16%)	20 (9%)
Not Done	11 (10%)	26 (24%)	37 (17%)

^a^Calculated by adding number of items from each site

#### Individual level

Overall, out of the 24 participants, 22 completed the program (87.5%), with completion defined as attending to at least six sessions of the workshop (Out of eight sessions). Fifty-four percent of participants attended all the sessions. Attendance per site was as follow: Site 1 had an average attendance of 90% per session (range 81–94%), while Site 2 had an average 82% attendance per session (range 67–89%). Reasons for non-completion were not collected.

When participants were asked to demonstrate the exercises 1 week after the completion of the program, 95% of them adequately demonstrated the exercises; however, only 57% of them were able to adequately explain the purpose of the exercise, even though it was explained during *Pisando Fuerte* sessions. Table 6 shows how many participants were using the behaviors learned in their daily lives 1 week after completing the program (fidelity of enactment).

**Table 6:** Fidelity of enactment & maintenance of behaviors 1 week after completion of the program (*n* = 18)

Behavior	Fidelity of enactment (1 week) *N* = 21	Maintenance (6 months) *N* = 18
Continued doing exercises regularly	14 (58%)	11 (58%)
Use of safe walking behaviors used	15 (75%)	17 (94%)
Use of safe measure to get up from a sitting position	15 (75%)	17 (94%)
Discussed with doctor or pharmacist medications that might increase risk of falls	1 (5%)	6 (35%)
Changes related to visual acuity (e.g. glasses, eye exam)	6 (33%)	5 (28%)
Environmental changes at home to decrease fall hazards	10 (50%)	12 (67%)
Changed footwear	9 (45%)	9 (50%)
Talked to a physician regarding falls or fall risk	7 (33%)	11 (61%)

### Maintenance

Table 6 shows the frequency of behaviors at 6 months after the program was completed. In the interviews with participant, when asked “What do you do differently to prevent falls since taking *Pisando Fuerte*?”, common themes were that participants were taking more time to get up from chairs, concentrating more on walking, walking more slowly, and being more aware of their surroundings when walking.

*“I pause for balance when standing up”*

*“Don't rush, take my time”*

*“The way I walk up and down stairs, more cautious and take my time.”*

Although maintenance was not evaluated at the organization level, a quote from a coordinator highlights the overall interest by their organization: “*This program is an education piece needed with seniors, especially for Spanish-speaking. It was well received, and participants feel so successful and pleased. Lack of funding would be a reason not to do it. As an agency, we are not set up to charge for programs. End result made all the frustration worthwhile.”*

## Discussion

This study shows that *Pisando Fuerte* significantly improves the behaviors that are associated with a decrease in the frequency of falls, as shown by the improvement of the FaB, and continued execution of exercises for balance and strength and safety strategies to prevent falls by older adults whose primary language is Spanish. It is important to note, our participants had low literacy levels, with 45% of participants having less than primary education, and 37.5% having difficulty reading in Spanish. This is substantially different from the experience with *Stepping On* participants, where participants with less than high school education are less than 20% [14, 21]. This highlights the need to perform more than translation, but the linguistic and cultural adaptation of the program.

Our study showed a promising reduction in falls (68%), which is similar to the results for *Stepping On* implementation, which have ranged between 31 and 48% [12, 13]; however, our results are not statistically significant because the sample size was not powered to identify a significant reduction in falls. Additionally, we collected two intermediate outcomes that are used as predictors of falls: FaB [24, 26] and TUG [23, 27]. Our study showed a significant improvement in FaB similar to other Stepping On studies [13]; however, TUG results had a non-significant trend to worsening, i.e. greater time required to execute the task. Several factors may explain the increased TUG times. First, many participants reported that as a result of this program they were “walking slower” and “taking their time” when changing positions, and per evaluator recollection, older adults were proud of doing tasks at a slower pace. With the TUG participants were told to “walk at a comfortable pace” similarly to other studies [23, 34]; we suspect that they may have slowed their walking with the TUG to demonstrate that they were now walking more safely. Other studies have used the TUG with instructions to walk “quickly” or “as quickly as you can in a safe way” [27]. We chose not to use these instructions as the focus of *Pisando Fuerte* is on walking more safely, including not walking too fast. In addition, some studies have also suggested that the TUG might not be a good tool to measure the risk of falling, because it might not have the sensitivity required to identify falls risk among older adults whose TUG scores are close to normal values [35, 36]. Lastly, there is limited information about the normative values of the test in Hispanic/Latinos populations in the US [37]. Regarding the uptake of protective health behaviors (Table 6), *Pisando Fuerte* can’t be compared with the original study published by Clemson et al. [12], because of the different methodologies used to evaluate the changes, per example, in our study maintenance was evaluated at 6-months, while Clemson et al. evaluated the changes at 14 months.

We identified several barriers for adoption by workshop leaders and organizations. First, Leaders were required to be fluent in English to complete the training, which narrows the number of individuals who could be trained to facilitate *Pisando Fuerte*. Second, the time required for preparing and delivering the program might be cost prohibitive for many organizations. Third, some of the materials in the leader manual were not translated into Spanish, and those that were translated were at too high of a language level for use with older adults with little formal education. As a result, leaders needed more preparation time to review the information provided, and adjust the language of the program as written in their leader manual to the language used by participants, especially around key words that have multiple variations across Latin America (e.g. calf muscle is known as *chamorro* in Mexico, *batata* in Puerto Rico, *pantorilla* in Venezuela, and *gemelos* in Panama and Central America).

Fidelity of delivery was evaluated following protocols established for Stepping On leaders [14]. For Stepping On it is expected that new leaders deliver at least 80% of elements in a satisfactory manner, however, this benchmark was established empirically from the identification of key elements [20, 21, 38]. In our study, the overall fidelity of delivery was below the pre-established benchmark of 80%, with substantial differences between sites, where site 1 had higher performance that site 2. We suspect that one of the reasons for the lower fidelity at site 2 was related to the leaders’ background, and the context (setting) where the classes were held. Site 1 had access to more resources based on the location of the workshops, the leaders had more experience facilitating group activities with Hispanic/Latino older adults, and the organization had experience delivering similar programs. Meanwhile, site 2 (senior housing building) had limited resources, one of the leaders was also the housing coordinator, which created logistical difficulties, and the organization had no previous experience in organizing this type of event. This correlates with the experience of Stepping On, where the implementation of the program in independent living facilities was cumbersome because the staff in charge of delivering the program were not given additional time or resources to implement the program, delivery of health promotion programs was not central to the organization’s mission [14].

Although most of the leaders had previous experience in teaching health education classes, not all of them had been trained in health promotion and behavior change techniques, which affected the delivery of the program, and potentially influenced the seniors’ adherence to the program, and their uptake of the protective behaviors Another important aspect to consider, is the familiarity that the leaders had with the participants. According to our previous experiences, when the leader is a recognized local champion, it facilitates recruitment to the program and subsequent adherence.

Because of the small number of participants per site, it is not possible to assess if the health outcomes, and uptake of health behaviors were affected by the lapses in fidelity, or by the backgrounds of participants (Table 2). Compared to site 1, participants in site 2 were more likely to be male, with low educational attainment and living alone, which is in itself different than the typical participant in a fall prevention program: white older female with some college education [39].

These differences may have affected adherence and uptake and maintenance of behavior change, highlighting the importance of future research studies that assess how participant characteristics may affect outcomes of falls prevention programs, particularly in the setting of cultural adaptation, where adaptations must take into account cultural and socio-demographics differences of the end-users.

Our study population is also different from the original Stepping On, in that our participants had a lower rate of falls at baseline. Clemson et al. [12] described that *Stepping On* is more effective among those with had multiple falls in the previous year. Future research studies will be powered to assess the effectiveness of *Pisando Fuerte* in fall rate, and other health outcomes.

The process of linguistic and cultural adaptation of *Stepping On* to *Pisando Fuerte* was performed in a previous study, and not described at length in this paper. However, it was characterized by the inclusion of community advisors, older adult stakeholders, leaders, and experts in the field in a process of iterative adaptations. Our study did not assess the cultural adaption per se, but rather the fidelity of delivery for cultural adaption elements, which was low. We hypothesize that this might be a result of the evaluation tool used. As seen in Table 3, leaders needed to refer to 6 different cultural values during session-3 to obtain a “satisfactory” evaluation; however, there is no evidence that all the values need to be displayed to increase understanding. Based on the overall results of this pilot, we believe that we achieved our goal for cultural adaptation: to promote participation in the program and maintenance of the desired behaviors by appealing to cultural values that are delivered in an understandable way [18, 40].

The limitations of our study include the small sample size, and the low rate of falls at baseline which limits our capacity to assess the effect of the program on the rate of falls. However, it is important to acknowledge that our study was not designed to identify changes in physical performance measures to assess strength, balance and mobility; neither was it powered to identify a statistically significant reduction in falls. The purpose of this study was to provide information about the acceptability and feasibility of the program in real world-settings, the fidelity of delivery, and participant uptake and maintenance of behavior change elements, and the potential barriers that need to be addressed before the program can be packaged for dissemination. The results of our study provide information that organizations will need in the process of deciding if they will offer *Pisando Fuerte* or similar prevention initiatives. Additionally, our results provide evidence of the unique needs that need to be addressed when evaluating programs for Hispanic/Latino communities.

## Conclusions

In conclusion, *Pisando Fuerte* is a culturally acceptable program to prevent falls among Hispanic/Latinos communities that increases adoption of behaviors to prevent falls. It provides similar benefits as *Stepping On* [13, 14], even in light of bigger challenges that needed to be overcome to adopt and implement the program. Although *Pisando Fuerte* was well accepted by instructors and participants alike, more work is required to be able to disseminate the program, including: (1) Develop and implement the Spanish language training for leaders; (2) Improve training around elements that had poor fidelity during implementation; (3) Revise materials to assure that cultural adaptation of the program is relevant to a diverse group of Hispanic/Latinos individuals. The rigorous evaluation we conducted was essential to identifying the remaining gaps with program adaptation, and how they relate to the context where the program is implemented. Our findings support the value of conducting a rigorous evaluation of implementation metrics with any cultural adaptation of a behavior change intervention, to ensure that the adapted program has adequate adoption and maintenance, can be implemented with good fidelity and minimal barriers, and can retain effectiveness similar to the original program.

## Data Availability

The datasets during and/or analyzed during the current study available from the corresponding author on reasonable request.

## Abbreviations

FaB: Fall Behavioral Scale
RR: Relative risk
TUG: Timed up and go
US: United States of America
